# Morphological Evidence for Novel Roles of Microtubules in Macrophage Phagocytosis

**DOI:** 10.3390/ijms24021373

**Published:** 2023-01-10

**Authors:** Yoshika Seta, Kumpei Kawakatsu, Shiori Degawa, Toshiyuki Goto, Takahito Nishikata

**Affiliations:** 1 Frontiers of Innovative Research in Science and Technology (FIRST), Konan University, Minatojima-Minamimachi, Chuo-ku, Kobe 605-0047, Japan; 2 Laboratory Cellular Function Imaging, RIKEN Center for Biosystems Dynamics Research (BDR), Minatojima-Minamimachi, Chuo-ku, Kobe 605-0047, Japan

**Keywords:** microtubule network, phagocytosis, macrophage, F-actin, membrane ruffling, serum-derived macrophage activating factor, Ca^2+^ signaling

## Abstract

Although the phagocytic activity of macrophages has long been studied, the involvement of microtubules in the process is not well understood. In this study, we improved the fixation protocol and revealed a dynamically rearranging microtubule network in macrophages, consisting of a basal meshwork, thick bundles at the cell edge, and astral microtubules. Some astral microtubules extended beneath the cell cortex and continued to form bundles at the cell edge. These microtubule assemblies were mutually exclusive of actin accumulation during membrane ruffling. Although the stabilization of microtubules with paclitaxel did not affect the resting stage of the macrophages, it reduced the phagocytic activity and membrane ruffling of macrophages activated with serum-MAF, which induced rapid phagocytosis. In contrast, the destabilization of microtubules with nocodazole enhanced membrane ruffling and the internalization of phagocytic targets suggesting an inhibitory effect of the microtubule network on the remodeling of the actin network. Meanwhile, the microtubule network was necessary for phagosome maturation. Our detailed analyses of cytoskeletal filaments suggest a phagocytosis control system involving Ca^2+^ influx, the destabilization of microtubules, and activation of actin network remodeling, followed by the translocation and acidification of phagosomes on the microtubule bundles.

## 1. Introduction

Macrophages participate in immune responses and tissue homeostasis by phagocytosing pathogens and dying cells [1]. Thus, the activation of macrophage phagocytosis plays an important role in human health and the treatment of disease (e.g., tumor immunity, and chronic inflammatory and autoimmune diseases) [2]. Phagocytosis can be roughly divided into six stages: (1) the resting stage, when macrophages have a relatively low likelihood of phagocytosis; (2) the activated stage, when macrophages are activated by macrophage-activating factor (MAF) and other inflammatory signals for probing the phagocytic target; (3) the target recognition stage, when receptors bind to the phagocytic target, and their downstream signaling pathways are activated; (4) the membrane deformation stage, when pseudopod extension and membrane raffling occur via cortical actin network remodeling; (5) the phagosome formation stage, when a protruded membrane (called a phagocytic cup) covers the phagocytic target and fuses at the distal side; and (6) the phagosome maturation stage, when internalized phagosomes are transformed into phagolysosomes, and target particles are degraded [3,4].

Phagocytic events, such as the extension of pseudopods and lamellipodia and the formation of phagocytic cups and phagosomes, depend on the actin network [5]. The pivotal role of the actin network has been well studied. For example, membrane ruffling after MAF stimulation results from the local disruption of cortical F-actin filaments by ADF/cofilin family proteins, followed by actin re-polymerization by small GTPase, cdc42, and the actin nucleator Arp2/3 complex to form lamellipodia [6,7,8]. Target recognition and activation of signaling pathways depend on the confinement and diffusion of phagocytic receptors, which are dynamically regulated by the cortical actin network [9,10]. Actin-dependent membrane remodeling differs depending on the target type [11].

However, studies on the role of microtubules in phagocytosis are limited. A major function of microtubules in phagocytosis is vesicle transport, which supports phagosome maturation for target degradation [12]. For example, the molecular complex of Rab7, Rab7-interacting lysosomal protein (RILP), and the OSBP-related protein 1 long splice variant (ORP1L) on the late phagosome membrane is connected to a dynein motor protein to facilitate the inward movement of phagosomes in the last step of phagocytosis [13]. This inward movement depends on astral microtubules radially extending from the centrosome, Golgi complex, or endoplasmic reticulum [14]. Another microtubule function is the supply of plasma membrane for pseudopod elongation [15]. For example, Golgi- and vesicle-associated PKC-ε molecules are required for transporting transmembrane proteins and the plasma membrane to phagosomes along the microtubule [16].

There are two more cases for which a close relationship between microtubules and phagocytosis has been suggested. One is LC3-associated phagocytosis, which resembles autophagy [17]. Acetylated microtubules are required for the fusion of autophagosomes with lysosomes [18]. The other mechanism is NLRP3 inflammasome activation [19]. Paclitaxel induces augmentation of NLRP3 inflammasome activation in lipopolysaccharide (LPS)-primed macrophages through α-tubulin acetylation [20]. These reports are related to the innate immune response against microbial infections, and microtubule acetylation and the inflammatory response occurred at least several hours after the first phagocytosis event. Thus, acetylation of microtubules is not directly related to phagocytosis. 

Recently, we focused on macrophage activation via serum-derived macrophage-activating factor (serum-MAF [21]) and developed a novel and rapid assay system that can evaluate phagocytic activity at least 10 min after the start of phagocytosis [22]. Serum-MAF is human serum treated with β-galactosidase and sialidase and is suggested to have anti-angiogenic and tumor-killing activities through the activation of macrophages [23]. When serum-MAF was applied to THP-1 (human leukemia monocytic cell line)-derived macrophages, annexin A2 showed punctate cortical localization within 30 s in a Ca^2+^-dependent manner [24]. The rearrangement of the cortical actin network and membrane raffling became obvious, forming intricate stacks of lamellipodia, designated as “frill-like structures,” within 10 min [25]. This frill-like structure is responsible for the significant activation of phagocytosis within 10 min by achieving a high efficiency of target capture [26].

In this study, to determine the function of microtubules in the fast activation of phagocytosis, we carefully observed the morphology of microtubules and found a dynamically rearranging microtubule network, even in the resting stage of the macrophages. This microtubule network exclusively correlated with the cortical actin network. When macrophages were activated by serum-MAF, microtubule bundles in the cortical area became relatively thick and disappeared at the site of the frill-like structure. Paclitaxel treatment inhibited the internalization of beads in serum-MAF-activated macrophages, and nocodazole delayed phagolysosome maturation. These results suggest the importance of the dynamic coordination of microtubules and microfilaments for effective phagocytosis.

## 2. Results

### 2.1. Human and Mouse Macrophages Had a Conspicuous Tubulin Network

**Figure 1:**
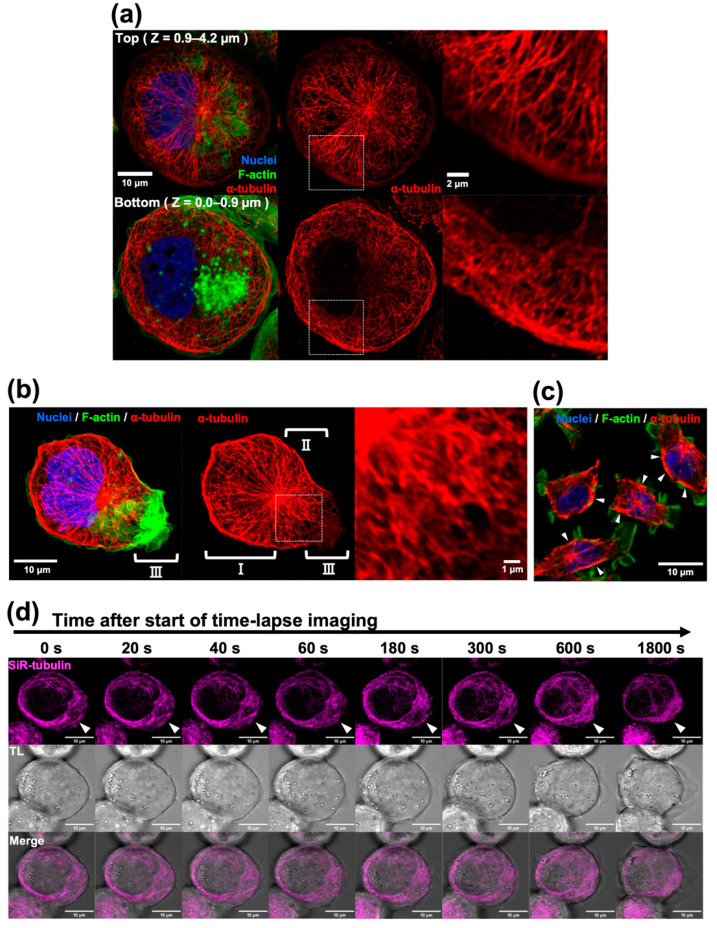
Microtubule staining of THP-1-derived macrophages. (**a**) Resting stage macrophages were fixed with a new protocol and immunostained with an anti-α-tubulin antibody (red), phalloidin (green), and DAPI (blue). Confocal optical sections of the upper half (Z = 0.9–4.2 µm) and lower half (Z = 0.0–0.9 µm) were stacked. The merged image (left), tubulin-single channel (middle), and enlarged image of the white boxed region (right) are shown. (**b**) The resting-but-somewhat-active macrophage was stained. The merged image (left), tubulin-single channel (middle), and enlarged image of the white boxed region (right) are indicated. The regions where the radial microtubules were evident (**I**), the pseudopod was protruded (**II**), and membrane ruffling was evident (**III**) are distinguished. (**c**) RAW-264.7 mouse macrophages were stained with an anti-α-tubulin antibody (red), phalloidin (green), and DAPI (blue). A single optical section of the merged image is shown. Arrowheads indicate that thick microtubule bundles covered microtubule-rich cytoplasm near the cell cortex. Notably, most of the cortical areas with thick microtubule bundles are free of membrane ruffling. Scale bars: 10 µm. (**d**) Serial images from a time-lapse movie of SiR-tubulin-stained resting macrophage are indicated. The SiR-tubulin-single channel (upper), the transparent light channel (TL; middle), and merged images (lower) are shown. Arrowheads indicate the tip of pseudopod protrusion and membrane ruffling. Just beneath the membrane ruffling, microtubules dynamically rearranged (for example, they disappeared as shown in (**b**), radially attached to the cortex, densely accumulated, or covered the microtubule-rich region). Some large vesicles are on the radial microtubule. Scale bars: 10 µm, 2 µm or 1 µm as indicated.

To assess the role of microtubules in phagocytosis, we improved our fixation protocol. Our new protocol preserves microtubule filaments and reduces background staining. As shown in Figure 1, in the bottom part of THP-1-derived macrophages, radial and arced microtubules formed a relatively uniform meshwork structure. The upper part of the macrophages contained a large nucleus. The centriole was situated in the cove-like pit of the nucleus and extended astral microtubules, some of which covered the nucleus and some ran underneath the cell membrane. Astral microtubules and the bottom meshwork appeared to be continuous or overlapped at the cell edge. These microtubules formed relatively thick bundles at the rim of the cells (Figure 1a).

Once the macrophage protruded a pseudopod and formed membrane ruffles, astral microtubules on the protruding side branched and distorted. Moreover, these microtubules are relatively thick and dense. Staining of the microtubule network and rim bundles became faint at the distal tip of the pseudopod. At the site of microtubule depolymerization, F-actin accumulated and formed membrane ruffles (Figure 1b). To determine whether the newly described microtubule network is a general structure, a mouse RAW 264.7 macrophage was fixed using our protocol and stained for microtubules and F-actin. Astral microtubules and thick microtubules at the cell edge were obvious. They extended membrane ruffles and lamellipodia with F-actin accumulation from the slit of the cortical microtubules or the microtubule-free area of the cell cortex (Figure 1c). The entire microtubule network was dynamically rearranged according to live imaging of SiR-tubulin-stained macrophages. In particular, in the lamellipodia-forming region, polymerization and depolymerization of the microtubules actively occurred (Figure 1d and Supplementary Figure S1).

### 2.2. The Microtubules Did Not Have A Great Impact on Phagocytosis in the Resting Stage

**Figure 2:**
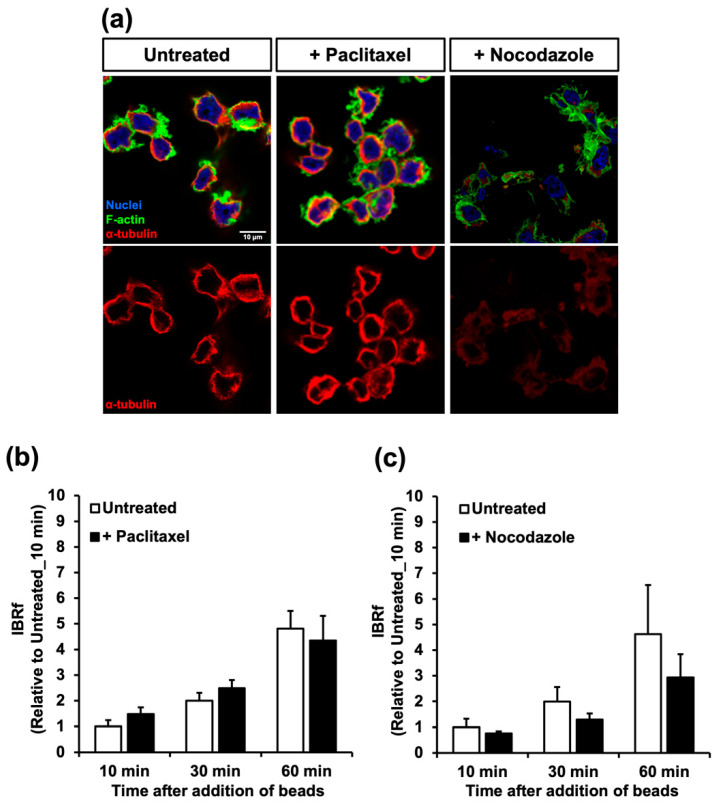
Effect of microtubule stabilization/destabilization on phagocytic activity. (**a**) THP1-derived macrophages treated with (+) paclitaxel or nocodazole and an untreated macrophage were stained with an anti-α-tubulin antibody (red), phalloidin (green), and DAPI (blue). Scale bars: 10 µm. (**b**,**c**) The phagocytic activities of the macrophages were measured as IBRf. Each bar indicates a value relative to that of the untreated control 10 min after the addition of the beads. Error bars represent standard deviations. At any time in this experiment, the phagocytic activities of the macrophages treated with these inhibitors were not significantly different from the concurrent untreated control group (ANOVA; n = 3, *p* > 0.05).

To determine the relationship between the microtubule network and phagocytosis, the macrophages were pre-incubated with paclitaxel (1h) or nocodazole (20 min) for stabilization or destabilization of the microtubules, respectively (Figure 2a). These drugs did not affect phagocytic activity for at least 1 h after pre-incubation (Figure 2b,c). Thus, the details of phagocytic activity were thoroughly analyzed using several parameters related to internalization, which were obtained from time-lapse recordings (Table 1). With paclitaxel, all of the internalization parameters (number of attached beads/cells, number of internalized beads/cells, and the division of these two numbers designated as phagocytic efficiency) for all of the duration periods of phagocytosis (i.e., 10, 30, and 60 min after bead addition) were similar to those of untreated macrophages. However, with nocodazole, all these parameters, except for the number of attached beads/cells at 10 min, were significantly increased compared to those of the control. Although nocodazole upregulated internalization, the phagocytic activity evaluated by the internalized bead ratio measured by fluorescence (IBRf) decreased. This may be caused by the process after internalization, which includes the maturation of phagolysosomes. When acidification of the phagolysosome was slow, the value of IBRf increased slowly because of the dim fluorescence of the AcidiFluor ORANGE-NHS (AFO) beads. This possibility is examined later in this study.

### 2.3. The Change in the Microtubule Network after Macrophage Activation

**Figure 3:**
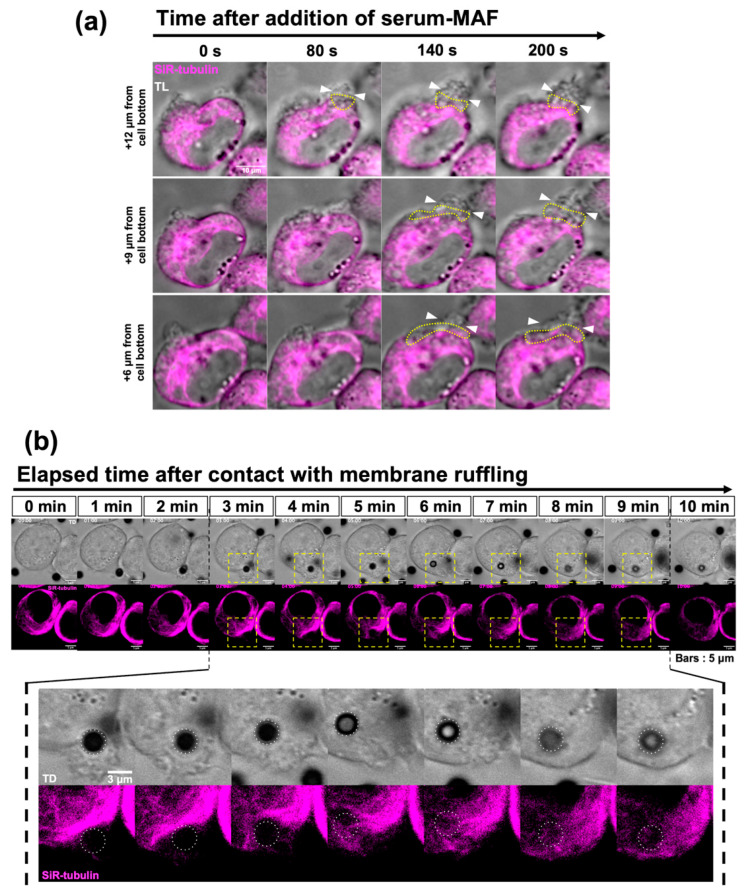
Effects of serum-MAF on phagocytosis and microtubules. (**a**) Changes in the microtubules just after serum-MAF activation was recorded with SiR-tubulin-stained macrophage. Merged images of SiR-tubulin (magenta) and transmitted light (grey scale) of different Z-positions of the optical sections (+6, +9, and +12 µm from the cell bottom) at indicated times (0, 80, 140, and 200 s after the addition of serum-MAF) are shown. The root of membrane extrusion is indicated by two arrowheads. The microtubule-free region of the cytoplasm was encircled by a dotted line. Membrane extrusion was very dynamic. The membrane extrusion indicated in this figure was expanded from the upper region (+12 µm 80 s) to the lower region (+6 µm 200 s). According to this expansion, microtubule staining in the cytoplasm became faint underneath the membrane extrusion. (**b**) Time-lapse images showing pseudopod extrusion (Upper; TL) and microtubules (lower; SiR-tubulin) during bead internalization. The time after the focused bead (encircled by dotted line) attached to the elongated membrane ruffling are indicated. Enlarged images indicated in broken rectangles are shown. Scale bars: 10 µm or 3 µm as indicated.

As the microtubule network of macrophages is mutually exclusive with F-actin accumulation, we focused on serum-MAF, which can rapidly activate macrophages’ phagocytic activity by enhancing membrane ruffling within 5 min [25]. Using live imaging of the SiR-tubulin-stained macrophages activated by serum-MAF, dynamic rearrangement of the microtubules was observed beneath the site of frill-like structure formation (Figure 3a,b and Supplementary Figures S2 and S3). In the first step of frill-like structure formation, lamellipodia-like thin membrane extrusion occurs in a small area of the cell cortex. Then, these thin membrane extrusions enlarged in size and expanded in area. This active membrane ruffle had a frill-like structure. Immediately before membrane extrusion, the microtubules disappeared from the cortical region of membrane extrusion.

Moreover, after the frill-like structure caught the beads, they moved inward. The cytoplasmic region with relatively thick and dense microtubules was situated underneath the membrane extrusion. When the internalized beads reached this region, the microtubules distorted and disappeared according to the progression of the inward movement of the beads. After the beads were passed, the microtubules were reorganized. This process is very fast. In the case of Figure 3b, it took 7 min.

### 2.4. Paclitaxel Inhibited Frill-like Structure Formation and Phagocytosis

**Figure 4:**
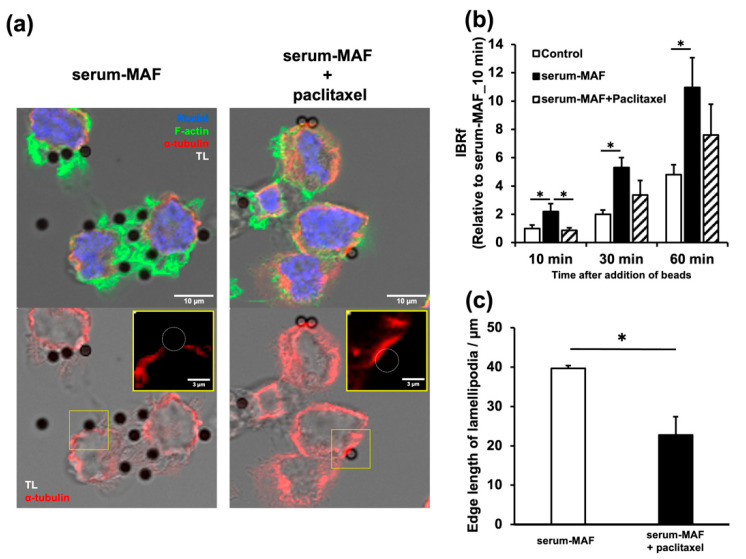
Effects of microtubule stabilization on phagocytosis. (**a**) Serum-MAF-activated macrophages pre-incubated with (right panel) or without (left panel) paclitaxel (10 µg/mL) for 60 min were incubated with beads for 10 min. A three-color image (upper) of α-tubulin (red), F-actin (green), and nuclei (blue) or a single-color image (lower) of α-tubulin (red) were merged with a grey scale of transmitted light (TL) image. Cortical microtubule staining was enhanced, and actin accumulation and bead attachment were inhibited by the paclitaxel treatment. Scale bar: 10 μm. A single red channel image of the boxed area was enlarged at the upper-right corner. The circle in the enlarged images indicate the invisible beads. Scale bar in the insert: 2 μm. (**b**) Phagocytic activity was evaluated by IBRf at 10, 30, and 60 min after bead addition. *; significantly different as determined by one-way ANOVA between the indicated groups (n = 3, * *p* < 0.05). On the contrary, the differences between the serum-MAF-activated macrophage and the serum-MAF-activated + paclitaxel macrophage at 30 and 60 min after bead addition were not significant (one-way ANOVA; n = 3, *p* = 0.091, *p* = 0.297, respectively). (**c**) Quantification of membrane ruffling. * *p* < 0.05, compared to the serum–MAF-activated control (t-test; n = 4).

As F-actin accumulation and the microtubule network are mutually exclusive, phagocytic activation by serum-MAF might be indirectly controlled by the microtubule network. Paclitaxel was administered to serum-MAF-activated macrophages (Figure 4). The stabilization of microtubules significantly inhibited the enhanced phagocytic activity evaluated with IBRf 10 min after bead addition (Figure 4a). Immunohistochemistry and quantification confirmed the stabilization of the microtubules, inhibition of frill-like structure formation, and a decrease in the number of attached beads after paclitaxel treatment (Figure 4b,c). Moreover, soon after the beads were attached to the cell membrane, cortical bundles of thick microtubules situated in the contact site disappeared in the control macrophages, whereas in the paclitaxel-treated macrophages, cortical microtubule bundles persisted at the contact site. (Figure 4b).

However, some internalization parameters and phagocytic activity up to 30 and 60 min were not inhibited (Table 2, Figure 4a). These results suggested that the mechanism of serum-MAF activation at 10 min is different from those at 30 and 60 min [24].

### 2.5. Depolymerization of the Microtubules Affected Phagolysosome Maturation

**Figure 5:**
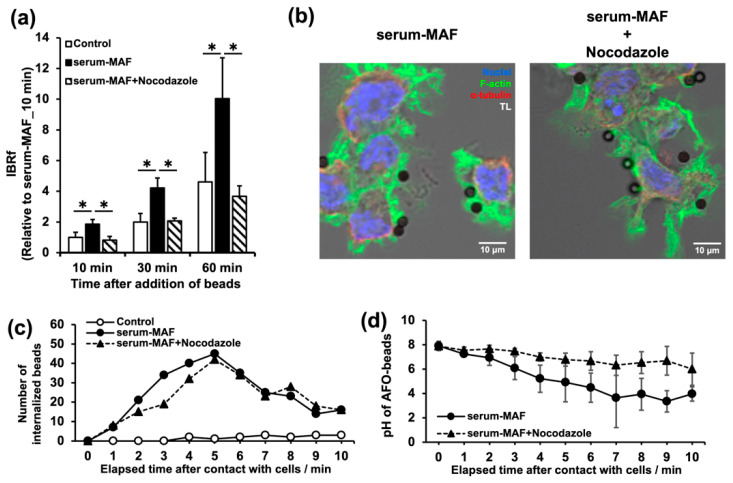
Effects of microtubule destabilization on phagocytosis. (**a**) Phagocytic activity was evaluated by IBRf at 10, 30, and 60 min after bead addition. *; significant as determined by one-way ANOVA between the indicated groups (n = 3, * *p* < 0.05). (**b**) Serum-MAF-activated macrophages pre-incubated with (right panel) or without (left panel) nocodazole (2.5 μg/mL) for 20 min were incubated with beads for 10 min. Three-color images of α-tubulin (red), F-actin (green), and nuclei (blue) were merged with a grey-scale transmitted light image. The membrane ruffles of nocodazole-treated macrophages were somewhat different from the typical frill-like structure. The extended membrane ruffles were relatively long and some of the lamellipodia were faintly stained with phalloidin suggesting that they were flat and thin. Although, the degree in membrane-ruffling extrusion was comparable to those without the nocodazole treatment. Scale bar: 10 μm. (**c**) The time required for bead internalization was measured in the same time-lapse recordings used for Table 3. As the phagocytic activity at 10 min after the addition of the beads was significantly increased in the serum-MAF-activated macrophage, the number of beads with an internalization time of less than 10 min was counted, and their frequency distribution was graphed. (**d**) The pH values of the internalized beads were measured according to the fluorescent intensity of the AFO beads every min after the beads were in contact with the cell surface. This measurement was also carried out with the same time-lapse recordings used for Table 3.

When nocodazole was applied to the serum-MAF-activated macrophages, the phagocytic activity evaluated with IBRf significantly decreased compared to the control level up to 60 min after bead addition (Figure 5a). However, the internalization parameters did not change (Table 3). Immunohistochemical staining of nocodazole-treated macrophages confirmed the depolymerization of microtubules and suggested that well-formed membrane ruffles, which were comparable to those in the controls, were responsible for the high internalization efficiency shown in Table 3 (Figure 5b).

**Figure 6:**
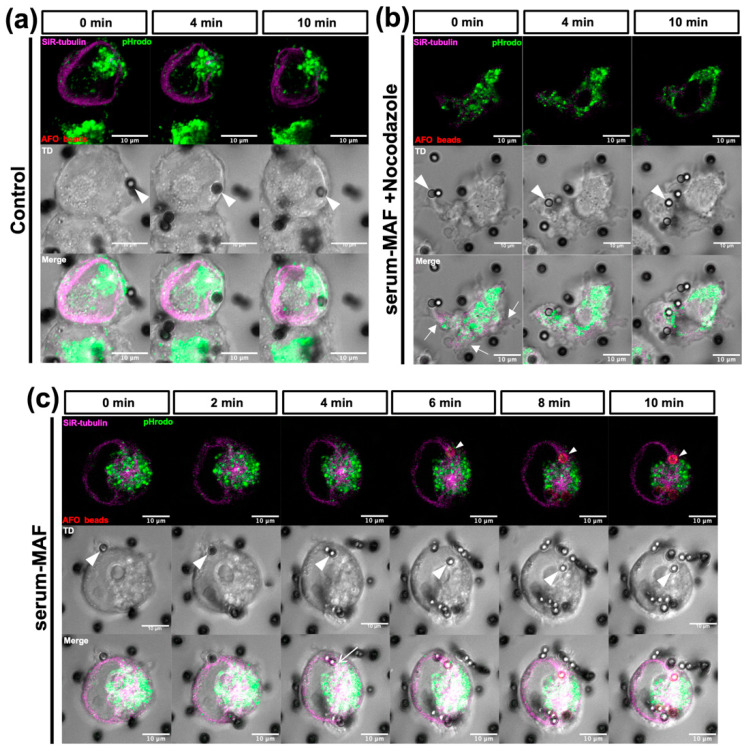
Translocation of the internalized beads and lysosome. Time-lapse recordings of macrophages stained with SiR-tubulin (magenta) for microtubules, pHrodo (green) for the lysosome, and AFO-beads (red) for acidification were conducted. Photographs at the indicated time periods after the bead of interest contacted the cell surface were selected from the movies. Beads of interest are indicated by arrowheads. (**a**) Control macrophage (without serum-MAF activation). The control macrophage, which internalized the beads at 4 min after the addition of the beads, was the rare and fastest case. The internalized bead was surrounded by the lysosome, while it had not yet acidified in 10 min. (**b**) Nocodazole and the serum-MAF-treated macrophage. The distorted cell shape with the elephant-nose-like cytoplasmic protrusion and extended flat and thin lamellipodia are shown (small arrows). (**c**) The serum-MAF-treated macrophage. The small lysosome was in contact with an internalized bead at 4 min in the serum-MAF-activated macrophage (arrow) and the bead started to emit fluorescence at 6 min (small arrowhead). Scale bars: 10 µm.

As nocodazole might affect internalization, we analyzed the speed of internalization and phagosome acidification from the same time-lapse recordings used for Table 3 (Figure 5c,d). The internalization time of the beads was measured retrospectively as the time from contact with the cell surface to complete internalization. The number of beads at each plot was counted as the beads that completed their internalization during each previous minute. Most of the serum-MAF-activated macrophages and serum-MAF-activated and nocodazole-treated macrophages were internalized within 10 min, and their average internalization times were 5.4 and 5.7 min, respectively, with a mode of 5 min (Figure 5c). Thus, nocodazole treatment did not affect the first half of the phagocytic process from probing to internalization. The drop in the pH of the beads was measured every minute after contact with the cell surface using AFO-beads (Figure 5d and Supplementary Figure S4). In serum-MAF-activated macrophages, acidification of phagosomes occurred continuously and reached a pH value of approximately 3.0, within 7 min. The nocodazole treatment delayed acidification, and the pH value of the internalized beads decreased only to 5.0 even 10 min after contact with the cell cortex. The effect of serum-MAF and nocodazole on phagosome maturation was observed by a time-lapse recording using AFO-beads, SiR-tubulin staining, and pHrodo staining of the lysosomes (Figure 6 and Supplementary Figure S5). The serum-MAF-activated macrophages showed faster internalization of the beads within 3 min after contact with the cell cortex compared to the control macrophages, in which internalization took more than 10 min. Moreover, from 4 min after contact with the cell cortex, the lysosomes were fused to the internalized beads, and from 6 to 10 min, and the beads were rapidly acidified and became phagolysosomes. During this acidification process, the internalized beads were translocated inward along the thick microtubule bundle extending from the centrosome to the cortical site, where internalization occurred.

In contrast, nocodazole-treated serum-MAF-activated macrophages showed distorted cell shapes and extended relatively fewer but long ruffles. These morphologies might support the idea that the microtubule network in macrophages has a restrictive role in the active actin network rearrangement. Although internalization of the beads occurred rapidly within 4 min and the lysosomes were situated near the internalized beads, no obvious fusion of the lysosomes, no active inward translocation of the beads, nor acidification of the phagosomes occurred. These results suggest that radially extended microtubules are necessary for the inward transport of phagosomes, the fusion of lysosomes to phagosomes, and the acidification of phagosomes. These events are important for phagosome maturation.

## 3. Discussion

Our new fixation protocol aimed to minimize the depolymerization of microtubules and extract cytoplasmic components, which could increase background staining. Since we have previously compared and examined fixation protocols for clear visualization of microtubules in another experimental system [27,28], we could improve the fixation and staining protocols. In some previous studies from other groups, although the authors did not mention the microtubule networks, there are clear photographs showing the microtubule network in macrophages, similar to our results [29,30]. Analyses of macrophages using innovative imaging technologies are becoming increasingly important for a precise understanding of immunology.

In this study, we first described the distribution of the intricate microtubule network in macrophages and its importance in phagocytosis. This microtubule network consists of a basal meshwork, thick bundles at the cell edge, and astral microtubules. Among them, the basal meshwork, bundles in the cell edge, and some of the astral microtubules extending beneath the apical cell membrane were present in the cell cortex. The cortical microtubule is not a common structure in ordinal cells except for epithelial cells, which have a cortical array of microtubules from the apical to the basal side nucleated from the CAMSAP (calmodulin-regulated spectrin-associated protein)-containing protein complex [31]. Cortical microtubules in epithelial cells are thought to be responsible for their columnar shape; thus, they are relatively stable structures [32]. In contrast, the macrophage microtubule network was rearranged dynamically, and microtubules in the apical cortex mainly extended from the centrosome. As the distribution of the microtubule network of macrophages was unique, its functions and regulatory mechanisms are intriguing.

The involvement of microtubules in macrophage phagocytosis can be divided into two phases: the internalization phase (including activation, target recognition, membrane deformation, and phagosome formation) and the phagosome maturation phase (including lysosome distribution, phagosome translocation, phagosome and lysosome fusion, and phagolysosome acidification). During the internalization phase, membrane ruffling, achieved by the dynamic rearrangement of the actin network, is important. The mutually exclusive expression patterns of microfilaments and microtubules suggest an intimate relationship between them. However, the effect of the destabilization of microtubules by paclitaxel did not affect either the membrane ruffling nor the phagocytic activities of the resting stage of the macrophages, while it affected serum-MAF-activated macrophages. This result did not support the idea that microtubules directly control phagocytic activities, while activation by serum-MAF is thought to be regulated by the microtubule network via controlling membrane ruffling. In our previous report, we reported that Ca^2+^ influx is involved in the formation of intricate membrane ruffling, designated as a frill-like structure, in serum-MAF-activated macrophages [24]. Thus, destabilization of microtubules by Ca^2+^ influx may be a key event in serum-MAF activation.

When the microtubules of the resting macrophages were depolymerized by nocodazole treatment, although the phagocytic activity was not affected, membrane ruffles were extended more widely (Figure 2a) and all of the bead internalization parameters were greater than those of the untreated control (Table 1). On the other hand, when the serum-MAF-activated macrophage was treated with nocodazole, internalization events including the formation of membrane ruffles comparable to the nocodazole-untreated control (Table 3, Figure 5b and Figure 6b) were not affected. These results suggested that the destabilization of the microtubule triggered membrane ruffling and phagocytic activation. They also suggested the inhibitory effect of microtubules on actin network remodeling and membrane ruffling. A similar regulatory effect of microtubules has been reported by Pineau et al. (2022) [33]. They demonstrated that microtubules and the GEF–H1–RhoA axis had an inhibitory effect on actin polymerization at the lymphocyte–antigen contact site.

For phagosome maturation, astral microtubules are essential for phagosome translocation and acidification by the fusion of the lysosomes [12,13]. In this study, some movies could represent the process of translocation, lysosome fusion, and the acidification of phagosomes in the radial microtubules. All of these events were inhibited by nocodazole treatment, suggesting a positive role for microtubules in phagosome maturation. In contrast, activation with serum MAF accelerated all of these events of phagosome maturation. These results highlight the uniqueness of the macrophage activation mechanism in serum MAF.

The inward movement of the phagosome on the microtubules is based on the minus-end-directed motor protein dynein. The speed of the inward movement of phagocytosed beads in serum-MAF-activated macrophages was roughly estimated from time-lapse movies. It was approximately 18.6 nm/s. It was close to the previously estimated speed of the dynein motor, 19 nm/s [34]. According to the mRNA expression profiles of the THP-1-derived macrophage [35], the corresponding motor is the commonly expressed cytoplasmic dynein 1, which consists of DYNC1H1, DYNC1I2, DYNLL1, and DYNLT1. As the commonly expressed dynein complex was used in the THP-1-derived macrophage and its moving speed was not accelerated, other events, such as lysosome fusion, might be responsible for the acceleration of phagosome maturation by serum-MAF activation.

Paclitaxel is a widely used anticancer drug, and its effect on cancer immunity has a certain impact on cancer therapy. According to our results, as the stabilization of microtubules had an inhibitory effect only on the rapid phagocytic activity of the macrophages, the effect of paclitaxel on cancer immunity appeared to be limited. Although, the more long-term effect of microtubule stabilization on macrophages’ activities is unclear. For example, paclitaxel-treated macrophages downregulated cell surface colony-stimulating factor 1 receptor (CSF-1R) and they were activated phagocytosis [36]. Thus, more studies on the role of microtubules for macrophage activation are needed to reveal the therapeutic mechanism of anticancer chemotherapy.

## 4. Materials and Methods

### 4.1. Reagents

The following reagents were purchased from the indicated sources: anti-α-tubulin antibody (clone DM1A), 12-O-tetradecanoyl-13-acetate (TPA), paclitaxel, and nocodazole (Sigma-Aldrich, St. Louis, MO, USA); 4′,6-diamidino-2-phenylindole dihydrochloride (DAPI; Nacalai Tesque, Kyoto, Japan); Dynabeads^®^ Protein G, Alexa Fluor™ Plus 647-conjugated anti-mouse IgG antibody, Alexa Fluor™ Plus 555-conjugated anti-mouse IgG antibody, Alexa Fluor™ 488-conjugated phalloidin, and pHrodo™ Green Zymosan A BioParticles™ Conjugate (Invitrogen, Oslo, Norway); AcidiFluor™ ORANGE-NHS (AFO: Goryo Chemical, Hokkaido, Japan); formaldehyde (Polysciences, Warrington, PA, USA).

### 4.2. Cell Culture

The THP-1 and RAW 264.7 cell lines were purchased from RIKEN BRC (Tsukuba, Japan). The THP-1 derived macrophage was obtained by incubation with 400 ng/mL TPA for 24 h.

### 4.3. Fixation and Immunofluorescence

The macrophages were fixed with seven volumes of fixation buffer (1.6% formaldehyde, 100 mM HEPES pH 7.0, 50 mM EGTA, 10 mM MgSO_4_, and 525 mM sucrose) for 30 s at approximately 20 °C. Then, three volumes of extraction buffer (0.5% Triton X-100 containing a fixation buffer) were added and incubated for 30 min with gentle agitation. All of the fixed samples were washed with PBS and immediately subjected to the staining step. To observe the microtubule network, fixed cells were immunostained with an anti-α-tubulin antibody (1:100) and Alexa Fluor™ Plus 555- or 647-conjugated secondary antibodies (1:1000). The cells were counterstained with 0.5 U/mL Alexa Fluor™ 488-conjugated phalloidin and 5 µg/mL).

### 4.4. Confocal Microscopy

For the time-lapse recording, the macrophages were stained with a 1 μM SiR-tubulin (microtubule-binding drug, Docetaxel, conjugated with silicon rhodamine) kit with or without 500 μg/mL pHrodo™ Green Zymosan A BioParticles™ Conjugate for 1 h. SiR-tubulin is an established fluorogenic probe that displays minimal toxicity in live-cell experiments [37]. Inhibitors were added during staining. The stained cells were washed with serum-free RPMI medium and immediately transferred for time-lapse recording. Immediately before recording, serum-MAF and/or AFO (AcidiFluor™ ORANGE) beads were added to the stage of the A1RHD25 confocal microscope (Nikon, Tokyo, Japan). Recording intervals were 20 or 60 s for the two-color or four-color images, respectively. The fixed specimens were observed under the same confocal microscope.

### 4.5. Phagocytosis Assay

Phagocytic activity was assayed according to a previously described method [22]. The cells were cultured in 96-well plates and treated with or without 8 μg/mL serum-MAF for 5 min. After that, the cells were washed with 1× PBS, and the culture medium was immediately replaced with serum-free medium containing 6 µg/well of the target beads. The target beads emitted red fluorescence due to the lower pH of the phagolysosome, since they were magnetic beads (Dynabeads^®^) labeled with AFO (AcidiFluor™ ORANGE). Ten, 30, and 60 min after the addition of the beads, the phagocytosed beads were measured using a fluorescence plate reader (TECAN, Männedorf, Switzerland). The phagocytic activity of the macrophages was evaluated as IBRf, which was calculated using the following formula:

$$\mathrm{IBRf}\;\left(\%\right)=\frac{\mathrm{fluorescence}\;\mathrm{intensity}\;\mathrm{of}\;\mathrm{internalized}\;\mathrm{beads}}{\mathrm{fluorescence}\;\mathrm{intensity}\;\mathrm{of}\;\mathrm{all}\;\mathrm{beads}}\times 100$$

### 4.6. Inhibitor Experiments

The macrophages were pre-incubated for 60 min with a microtubule-stabilizing drug, paclitaxel (10 µg/mL), or for 20 min with a microtubule-destabilizing drug, nocodazole (2.5 µg/mL). For serum MAF activation, serum MAF (8 µg/mL) was added during the last 5 min of each pre-incubation period. After that, the cells were quickly washed with PBS and replaced with a serum-free medium containing 6 µg/well of pH-sensitive beads.

### 4.7. Statistical Analysis

Unless otherwise indicated, data are presented as the mean ± SD of at least three independent experiments. The number of cells assessed in the quantitative analysis of a number of contacted/internalized beads is indicated within the table legends. All of the statistical analyses were performed using Microsoft^®^ Excel. Unless otherwise indicated, the two-tailed Student’s t-test was used to compare the two conditions. Differences between the groups in the IBRf measurement data were compared by one-way analysis of variance (ANOVA). Significance was determined as *p* < 0.05.

## 5. Conclusions

The intricate and dynamic microtubule network in macrophages was described for the first time in this study. The microtubule network regulates the entire process of phagocytosis. The inhibitory effect of microtubules on actin-network-controlled membrane ruffling and the internalization of targets was demonstrated. During phagosome maturation, microtubules affect the translocation of phagosomes and lysosomes, the fusion of lysosomes with phagosomes, and the acidification of phagolysosomes. Elucidation of the role of the macrophage microtubule network may pave the way for effective cancer immunotherapy.

## Figures and Tables

**Table 1:** Effect of microtubule stabilization/destabilization on bead internalization.

	(1) Untreated ^§^	(2) +Paclitaxel ^¶^	(3) +Nocodazole ^†^	(2)/(1)	(3)/(1)
up to 10 min					
Number of attached beads/cells	1.46 ± 0.22	1.48 ± 0.16	1.85 ± 0.25	1.01	1.27
Number of internalized beads/cells	0.15 ± 0.07	0.11 ± 0.03	0.33 ± 0.01 *	0.73	2.20
Phagocytic efficiency (%)	9.88 ± 2.77	7.56 ± 2.61	17.76 ± 1.89 *	0.77	1.80
up to 30 min					
Number of attached beads/cells	1.65 ± 0.28	1.55 ± 0.16	2.31 ± 0.17 *	0.94	1.40
Number of internalized beads/cells	0.27 ± 0.07	0.25 ± 0.06	0.76 ± 0.11 *	0.93	2.81
Phagocytic efficiency (%)	16.05 ± 1.83	16.73 ± 5.62	44.14 ± 13.42 *	1.04	2.75
up to 60 min					
Number of attached beads/cells	2.11 ± 0.44	1.75 ± 0.09	3.18 ± 0.24 *	0.83	1.51
Number of internalized beads/cells	0.44 ± 0.01	0.35 ± 0.07	1.03 ± 0.12 *	0.80	2.34
Phagocytic efficiency (%)	21.70 ± 5.01	19.87 ± 4.56	53.83 ± 19.13 *	0.92	2.48

§: 110 cells were analyzed, ¶: 128 cells were analyzed, †: 101 cells were analyzed, *: significantly different from serum-MAF treatment only (t-test, * p < 0.05).

**Table 2:** Effect of microtubule stabilization on the beads’ internalization process in the serum-MAF-activated macrophage.

	(1) serum-MAF ^§^	(2) serum-MAF+Paclitaxel ^¶^	(2)/(1)
up to 10 min			
Number of attached beads/cells	2.88 ± 0.37	1.37 ± 0.04 *	0.48
Number of internalized beads/cells	2.39 ± 0.30	0.16 ± 0.04 *	0.07
Internalization efficiency (%)	84.76 ± 0.86	11.52 ± 3.02 *	0.14
up to 30 min			
Number of attached beads/cells	5.08 ± 1.34	1.83 ± 0.14 *	0.36
Number of internalized beads/cells	3.59 ± 0.77	1.02 ± 0.20 *	0.28
Internalization efficiency (%)	71.46 ± 6.42	55.51 ± 7.62	0.78
up to 60 min			
Number of attached beads/cells	6.14 ± 1.45	2.96 ± 0.24	0.48
Number of internalized beads/cells	4.56 ± 0.74	1.73 ± 0.11 *	0.38
Internalization efficiency (%)	75.48 ± 5.36	58.73 ± 1.65	0.78

§: 119 cells were analyzed, ¶: 141 cells were analyzed, *: significantly different from serum–MAF treatment only (t-test, * p < 0.05).

**Table 3:** Effect of microtubule destabilization on the bead’s internalization process in the serum-MAF-activated macrophage.

	(1) serum-MAF ^§^	(2) serum-MAF+Nocodazole ^¶^	(2)/(1)
up to 10 min			
Number of attached beads/cells	2.88 ± 0.37	3.00 ± 0.09	1.04
Number of internalized beads/cells	2.39 ± 0.30	2.34 ± 0.22	0.98
Internalization efficiency (%)	84.76 ± 0.86	77.96 ± 5.35	0.92
			
up to 30 min			
Number of attached beads/cells	5.08 ± 1.34	5.91 ± 0.89	1.16
Number of internalized beads/cells	3.59 ± 0.77	3.96 ± 0.42	1.1
Internalization efficiency (%)	71.46 ± 6.42	67.60 ± 5.08	0.95
			
up to 60 min			
Number of attached beads/cells	6.14 ± 1.45	6.91 ± 1.19	1.13
Number of internalized beads/cells	4.56 ± 0.74	4.92 ± 0.73	1.08
Internalization efficiency (%)	75.48 ± 5.36	71.70 ± 4.16	0.95

§: 119 cells were analyzed, and ¶: 110 cells were analyzed.

## Data Availability

The data presented in this study are available in the Supplementary Materials.

## Supplementary Materials

The following supporting information can be downloaded at: https://www.mdpi.com/article/10.3390/ijms24021373/s1.
