# Supplementary material for: Morphological Evidence for Novel Roles of Microtubules in Macrophage Phagocytosis
Source: Int J Mol Sci. 2023 Jan 10;24(2):1373. doi: 10.3390/ijms24021373 (PMC9866147; doi:10.3390/ijms24021373)
Supplement: Supplementary file 1 [file ijms-24-01373-s001.zip › ijms-2112401-supplementary.pptx]

## Slide 1
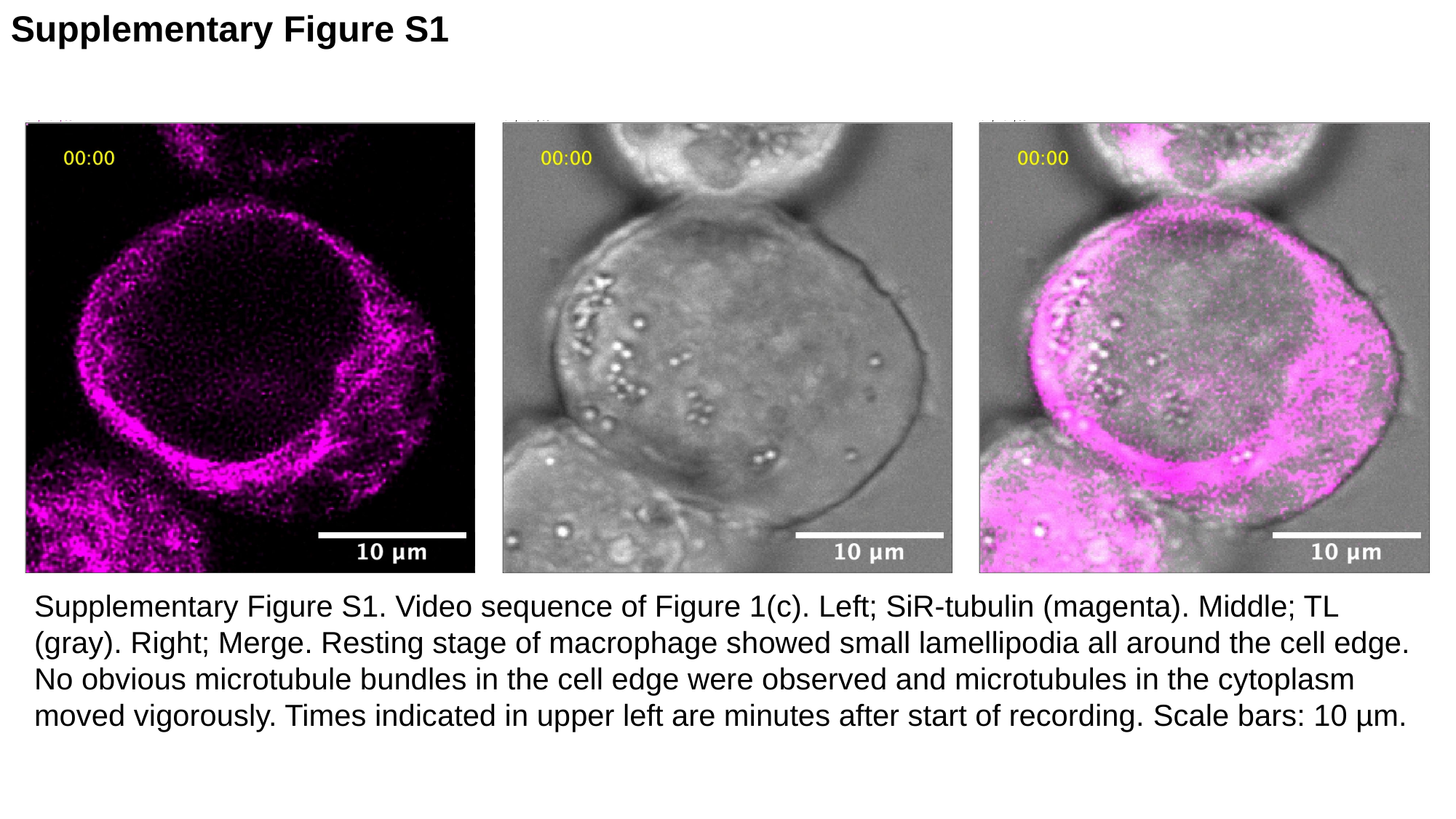

Supplementary Figure S1
Supplementary Figure S1. Video sequence of Figure 1(c). Left; SiR-tubulin (magenta). Middle; TL (gray). Right; Merge. Resting stage of macrophage showed small lamellipodia all around the cell edge. No obvious microtubule bundles in the cell edge were observed and microtubules in the cytoplasm moved vigorously. Times indicated in upper left are minutes after start of recording. Scale bars: 10 µm.

## Slide 2
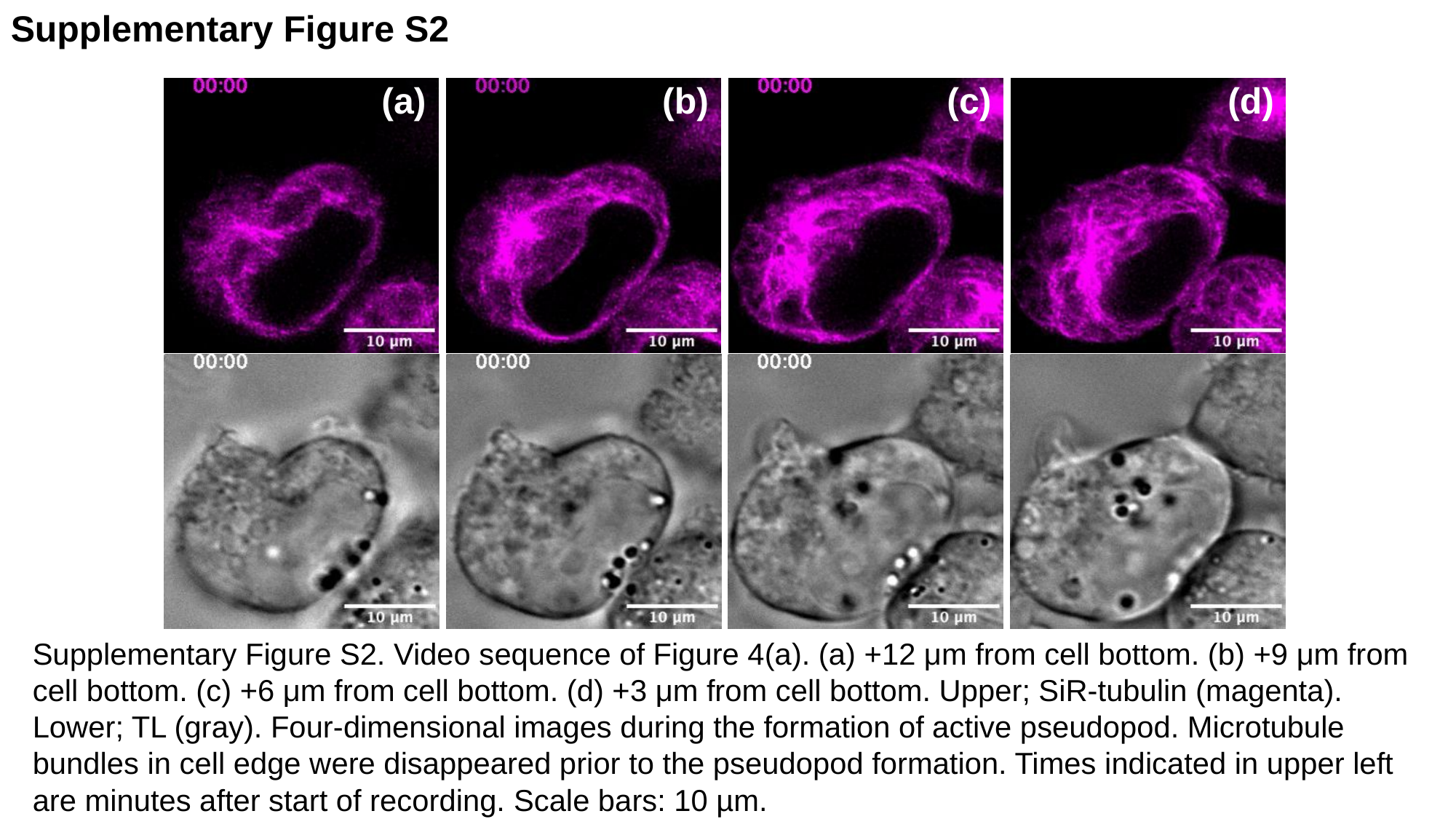

Supplementary Figure S2
(a)
(b)
(c)
(d)
Supplementary Figure S2. Video sequence of Figure 4(a). (a) +12 μm from cell bottom. (b) +9 μm from cell bottom. (c) +6 μm from cell bottom. (d) +3 μm from cell bottom. Upper; SiR-tubulin (magenta). Lower; TL (gray). Four-dimensional images during the formation of active pseudopod. Microtubule bundles in cell edge were disappeared prior to the pseudopod formation. Times indicated in upper left are minutes after start of recording. Scale bars: 10 µm.

## Slide 3
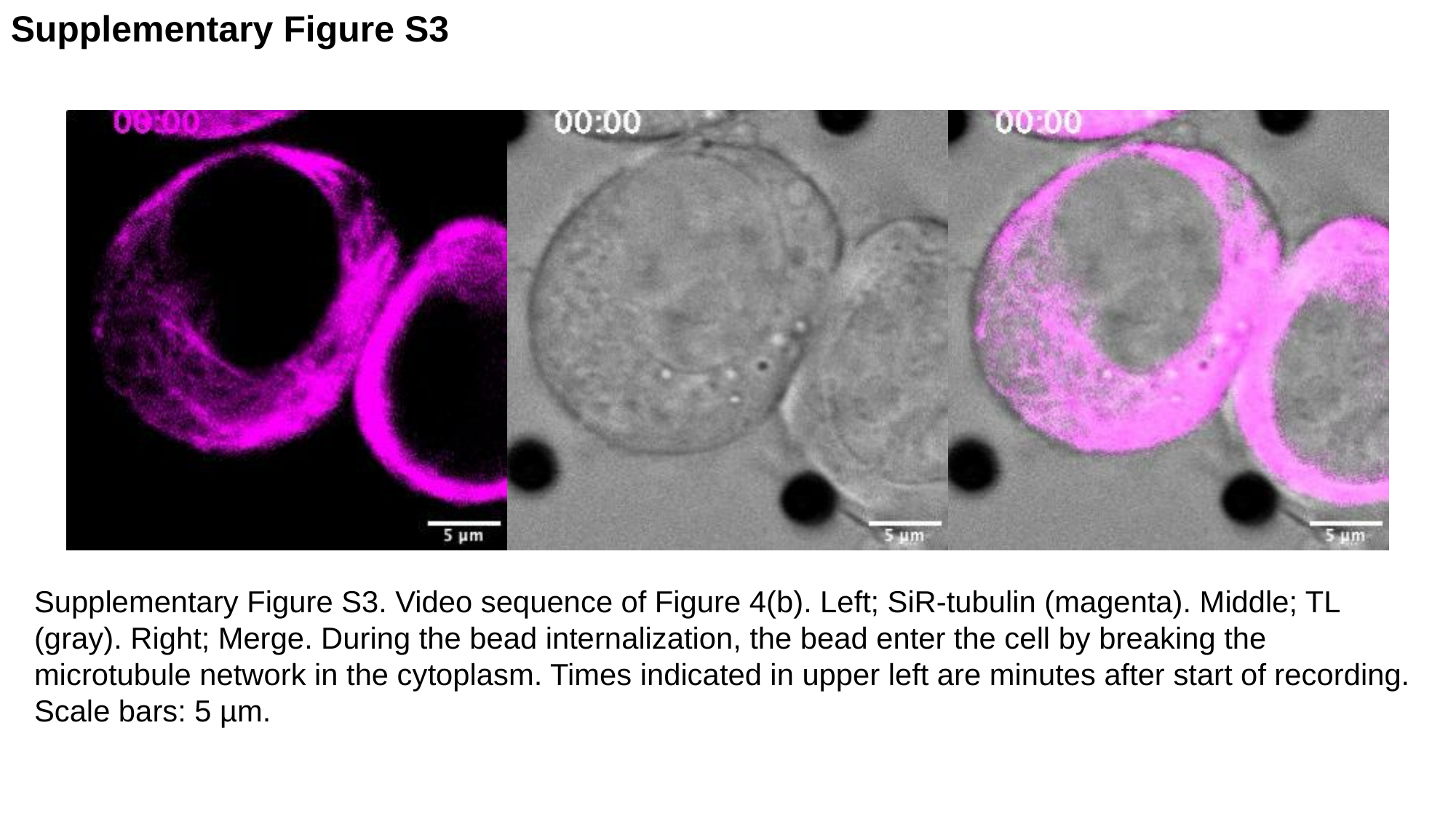

Supplementary Figure S3
Supplementary Figure S3. Video sequence of Figure 4(b). Left; SiR-tubulin (magenta). Middle; TL (gray). Right; Merge. During the bead internalization, the bead enter the cell by breaking the microtubule network in the cytoplasm. Times indicated in upper left are minutes after start of recording. Scale bars: 5 µm.

## Slide 4
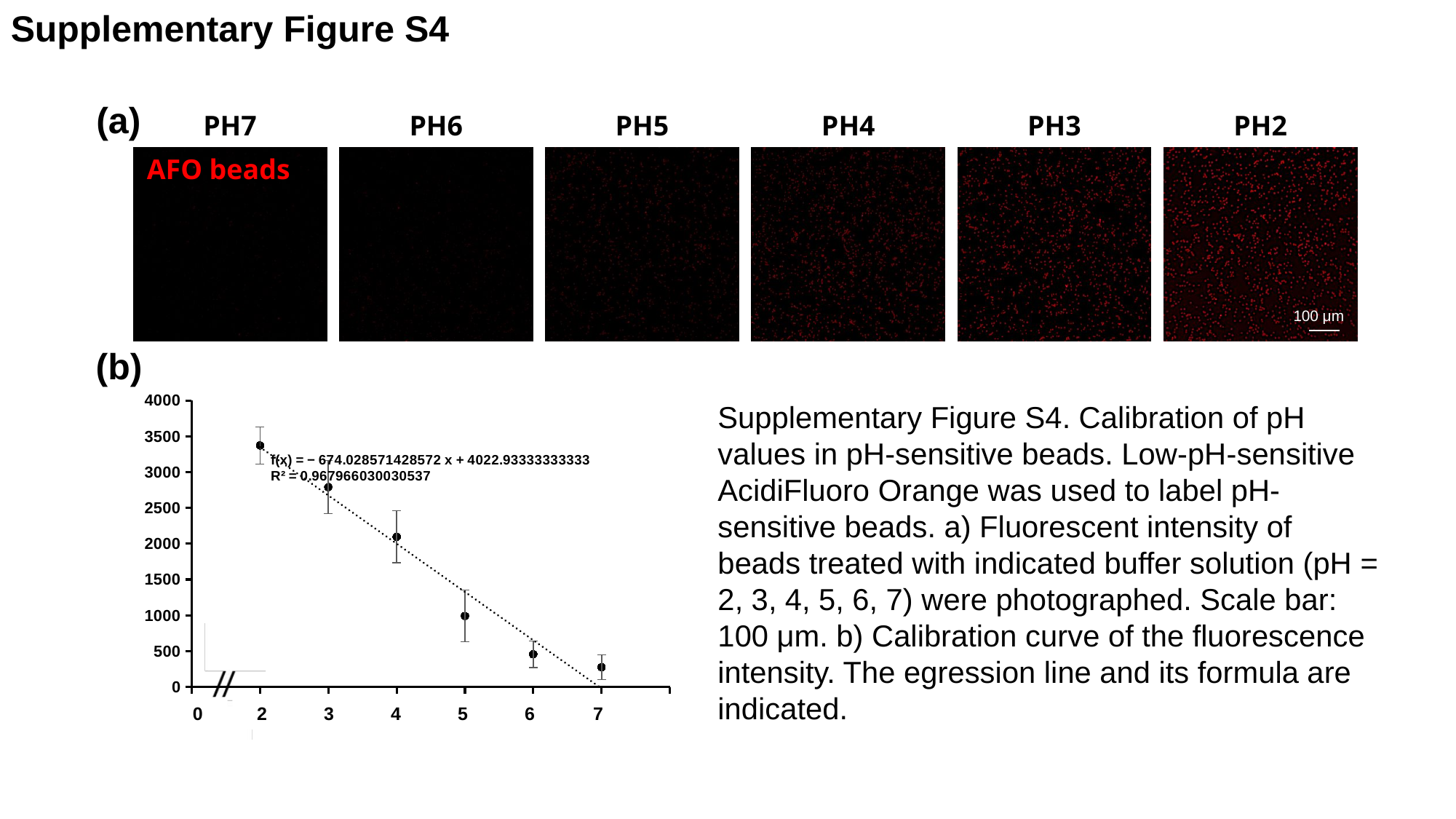

Supplementary Figure S4
(a)
PH7
PH6
PH5
PH4
PH3
PH2
AFO beads
100 μm
(b)
### Chart
| Category | F.I. |
|---|---|
0
2
3
4
5
6
7
Supplementary Figure S4. Calibration of pH values in pH-sensitive beads. Low-pH-sensitive AcidiFluoro Orange was used to label pH-sensitive beads. a) Fluorescent intensity of beads treated with indicated buffer solution (pH = 2, 3, 4, 5, 6, 7) were photographed. Scale bar: 100 μm. b) Calibration curve of the fluorescence intensity. The egression line and its formula are indicated.

## Slide 5
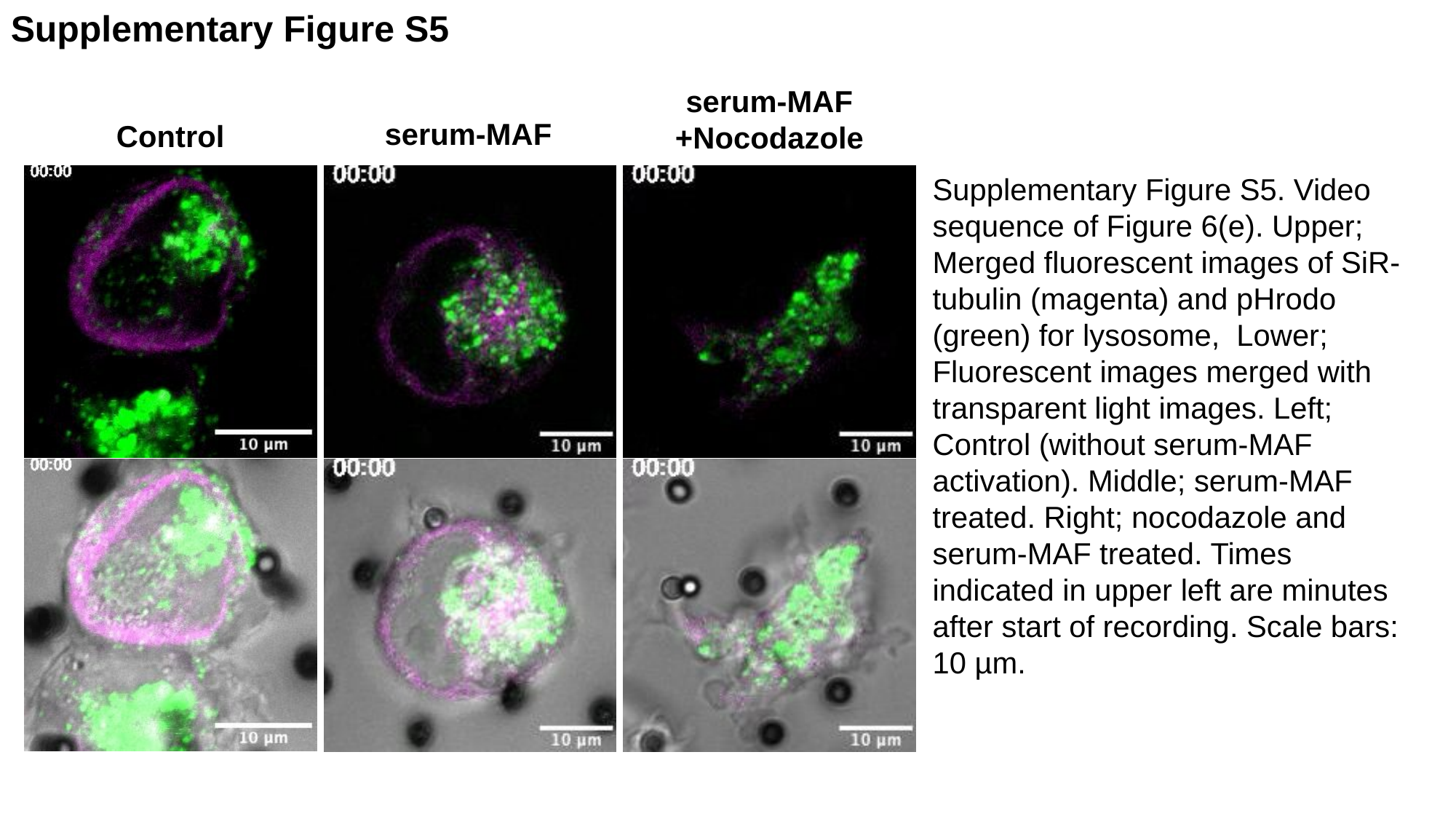

Supplementary Figure S5
serum-MAF
+Nocodazole
serum-MAF
Control
Supplementary Figure S5. Video sequence of Figure 6(e). Upper; Merged fluorescent images of SiR-tubulin (magenta) and pHrodo (green) for lysosome, Lower; Fluorescent images merged with transparent light images. Left; Control (without serum-MAF activation). Middle; serum-MAF treated. Right; nocodazole and serum-MAF treated. Times indicated in upper left are minutes after start of recording. Scale bars: 10 µm.
